# A Study on the Changing Law of Bacterial Communities in the Milk of Bactrian Camels with Subclinical Mastitis

**DOI:** 10.3390/microorganisms13061394

**Published:** 2025-06-15

**Authors:** Wanpeng Ma, Lin Zhang, Huaibing Yao, Yi Zhang, Wei Wang, Yifan Liu, Xueting Zhao, Zhanqiang Su

**Affiliations:** 1 College of Veterinary Medicine, Xinjiang Agricultural University, Urumqi 830052, China; 320220066@stu.xjau.edu.cn (W.M.); 320230074@stu.xjau.edu.cn (L.Z.); 120130015@xjau.edu.cn (Y.Z.); 320232873@stu.xjau.edu.cn (W.W.); 320232797@stu.xjau.edu.cn (Y.L.); 320242918@stu.xjau.edu.cn (X.Z.); 2 Xinjiang Key Laboratory of New Drug Research and Development for Herbivorous Animals, Urumqi 830052, China; 3 Xinjiang Laboratory of Special Environmental Microbiology, Institute of Microbiology, Xinjiang Academy of Agricultural Sciences, Urumqi 830091, China; yaohuaibing@stu.xju.edu.cn

**Keywords:** Bactrian camel, subclinical mastitis, pathogenesis, 16S rRNA amplicon sequencing, camel milk bacterial community

## Abstract

Subclinical mastitis is a critical disease affecting camel health and milk quality. However, research on shifts in milk bacterial communities following subclinical mastitis in camels is limited. We evaluated changes in bacterial communities following subclinical mastitis in Bactrian camels. Three portions of California Mastitis Test (CMT)-negative milk and five portions of CMT-positive milk were collected from each Jimunai County and Keping County using the CMT, and the bacterial community composition of the camel milk was analyzed using amplicon sequencing of the v34 region of the 16S rRNA gene. Subclinical mastitis induced genus-level differences in the core bacterial microbiota of Bactrian camel milk. To our knowledge, *Delftia* was identified in camel milk for the first time, predominantly in Jimunai County. Bacterial abundance in camel milk from Keping County was increased and altered. Alpha diversity analysis revealed that subclinical mastitis induced lower and higher bacterial abundance in milk from Jimunai County and Keping County, respectively, compared to that of healthy camels. Therefore, these findings provide direction for future research on pathogenic microorganisms for the prevention and control of subclinical mastitis in Bactrian camels.

## 1. Introduction

Bactrian camels constitute a unique livestock resource in China and are predominantly found in the sparsely populated Gobi Desert regions of northwest China [1,2]. Data from the National Bureau of Statistics of China indicate that, as of 2023, the number of Bactrian camels in China had reached 561,800 animals, mainly concentrated in Xinjiang, Inner Mongolia, and Gansu, with populations of 315,600, 206,300, and 39,900, respectively. In Xinjiang, Bactrian camels are bred in areas such as Urumqi, Altay, Changji, Hami, and Hotan, most of which are situated in the desert and Gobi terrain. These camels provide vital daily necessities, such as milk, meat, and wool, for farmers and herders in pastoral regions [3], with camel milk serving as a crucial nutritional source for herders in these areas [4,5]. Compared to milk from other ruminants, camel milk has higher levels of immunoglobulins (Igs), lactoferrin, and calcium, along with lower fat content. It also contains various antibodies and bioactive proteins, including IgM, IgA, lactoferrin, lysozyme, lactoperoxidase, and *N*-acetylglucosaminidase [6]. Furthermore, camel milk is considered a special medicinal ingredient, known for its benefits in tonifying the spleen and qi, strengthening muscles and bones, calming nerves, nourishing yin, and detoxification. It is particularly effective for post-illness recovery and addressing overeating habits [5,7]. Camel milk is preferred among consumers owing to its exceptional medicinal and nutritional properties [8,9,10]. In addition, camel milk is extensively used in health maintenance as an alternative to infant formula milk powder and in the production of camel milk applications such as soaps and facial masks. This has resulted in an upsurge in demand for camel milk [6,11,12,13], fueling the rapid development of the camel milk industry and boosting global camel milk production at an annual growth rate of 2.45% [12]. Consequently, it has made significant contributions to global food security, nutritional security, economic growth, and poverty reduction efforts [4]. However, the high incidence of mastitis not only affects the healthy development of the camelid dairy industry but also has a huge impact on public health and safety [14,15].

Mastitis is an inflammatory condition affecting the mammary glands and primarily manifests in clinical and subclinical forms. Clinical mastitis presents with evident symptoms including breast swelling, congestion, fever, and pain. In contrast, subclinical mastitis is asymptomatic and can only be confirmed through testing, making it more prevalent than clinical mastitis [16]. The onset of mastitis can result in decreased camel milk production, spoiled camel milk, and increased treatment and labor expenses [17,18]. Currently, the average global incidence rate of dromedary camel mastitis is 45.66%; however, this rate varies significantly across different regions, with countries such as Israel (81.00%), Bangladesh (51%), Sri Lanka (49.00%), India (47.63%), Nigeria (40.40%), Jordan (31.40%), the United Arab Emirates (24.00%), and Somalia (16.66%) reporting cases of dromedary camel mastitis [17,19]. In contrast to dromedary camels, there are comparatively fewer reports of mastitis in Bactrian camels. To our knowledge, the incidence of clinical mastitis in Chinese Bactrian camels has not been reported; however, the incidence rate of subclinical mastitis ranges from 3.8 to 26.00% [20,21]. Numerous factors, including age, parity, lactation stage, season, and milking technique, contribute to camel mastitis. Bacterial infection of the mammary glands is the primary cause of mastitis [22], with *Staphylococcus aureus* (*S. aureus*) being identified as the predominant pathogen responsible for mastitis in dromedary camels. Furthermore, other pathogenic bacteria such as *Escherichia coli* (*E. coli*), *Streptococcus agalactiae* (*S. agalactiae*), various *Streptococcus species*, *Klebsiella pneumoniae* (*K. pneumoniae*), and *Pasteurella multocida* (*P. multocida*) can also induce mastitis [18,23,24,25,26,27]. Notably, 80% of acute gangrenous mastitis cases in the United Arab Emirates are attributed to *S. agalactiae* [28]. In Bactrian camels, mastitis is predominantly caused by pathogens such as *S. aureus*, *E. coli*, *Acinetobacter baumannii* (*A. baumannii*), *K. pneumoniae*, and *Enterococcus faecalis* (*E. faecalis*) [21].

Research on mastitis-causing pathogenic microorganisms has relied predominantly on traditional bacteriology. Nevertheless, milk harbors a diverse array of bacterial species, and selective culture media cannot be identified. Consequently, numerous unculturable bacteria remain undetected, leading to an inability to diagnose 25% of bacterial mastitis cases using bacterial culture methods [29,30,31]. Rapid advancements in molecular biology techniques have effectively addressed the deficiencies of traditional bacterial culture and disease diagnosis approaches [31].

Specifically, high-throughput sequencing technology has been used to analyze the microbial communities in milk and dairy products [32], with 16S rRNA gene amplicon sequencing being particularly prevalent in studies on bacterial communities across diverse ecosystems [33]. However, limited reports exist on the use of high-throughput sequencing technology to explore microbial communities in camel milk. Rahmeh et al. and Kadri et al. analyzed bacterial communities in dromedary camel milk [34,35], whereas Sun et al. and Qin et al. focused on bacterial communities in Bactrian camel milk [36,37,38]. These reports primarily focused on the variations in bacterial communities in fresh camel milk and identifying those associated with camel mastitis. Notably, Rahmeh R et al. exclusively used Illumina amplicon sequencing to examine the alterations in bacterial communities in dromedary camel milk during mastitis [31,39].

The present study aimed to reveal the impact of changes in bacterial communities on the occurrence of subclinical mastitis in Bactrian camels. To our knowledge, this study is the first to investigate the changes in bacterial communities in the milk of Bactrian camels with subclinical mastitis from different regions of Xinjiang using 16S rRNA gene amplicon sequencing. Our findings will potentially provide a basis for future research to prevent pathogenic infections during subclinical mastitis in Bactrian camels.

## 2. Materials and Methods

### 2.1. Experimental Animals and Specimen Collection

Bactrian camels selected from Jimunai and Keping Counties in Xinjiang, China, were used in this study. Situated in the northern section of the Xinjiang Uyghur Autonomous Region, Jimunai County spans from 47°01′ to 47°54′ N and 85°01′ to 87°02′ E. The terrain encompasses the Gobi Plains, low mountain/hill regions, mid-mountains, and high mountains. The highest extreme temperature recorded is 37.2 °C, while the lowest extreme temperature dropped to −38.8 °C. The county experiences an arid climate, with an average annual precipitation of 202.2 mm. From April to October each year, free-range grazing is the main practice, during which Bactrian camels feed on 127 types of wild forage. From November each year to March the following year, herdsmen mainly provide wild forage, alfalfa, corn straw, wheat straw, corn flour, and other supplementary feeds [40]. Keping County is situated in the southern region of Xinjiang Uyghur Autonomous Region, spanning from 39°51′ to 40°51′ N and 77°45′ to 79°57′ E. The terrain comprises both hills and plains, experiencing extreme temperatures ranging from a high of 43.1 °C to a low of −29.3 °C. It has a continental warm temperate arid climate, with an annual average precipitation of 73.8 mm. Owing to the limited grassland area, Bactrian camels are predominantly raised in captivity, with breeding enterprises or herders mainly feeding them alfalfa, corn stalks, cornmeal, and refined feeds.

The Bactrian camels selected for this study were all females, aged 7–8 years, with a lactation period of 2 months. The California Mastitis Test (CMT) and milk sample collection were conducted in May, and no other medical history was reported [19,41]. During milk sample collection, the udder was sterilized with 70% alcohol, and the first stream of camel milk was discarded. The collected milk samples were mixed with an equal volume of CMT diagnostic solution (Beijing Dingguo Changsheng Biotechnology Co, Ltd., Beijing, China), and gently swirled to evaluate the degree of gel formation. If the CMT slurry formed a gel-like consistency, the result was considered positive; if not, it is considered negative [42]. After testing, three CMT-negative camel milk samples were collected from Jimunai County and Keping County, and five CMT-positive camel milk samples were collected from each county. All female camels used for milk sample collection showed no clinical symptoms. A total of 10 mL of camel milk was collected from each Bactrian camel using sterile sampling bottles and transported to the laboratory for backup [31].

### 2.2. DNA Extraction, Polymerase Chain Reaction Amplification, and Sequencing for the v3–v4 Region of the 16S rRNA Gene

Using the CTAB method to extract bacterial genomic DNA, first, 1000 µL of CTAB lysate (Beijing Dingguo Changsheng Biotechnology Co, Ltd., Beijing, China) was added to a 2.0 mL EP tube, followed by lysozyme (Beijing Dingguo Changsheng Biotechnology Co, Ltd., Beijing, China) Then, 500 µL of the milk sample was added to the lysate and preheated in a 65 °C water bath for 30 min with gentle shaking. The mixture was then centrifuged at 12,000 rpm for 10 min to obtain the supernatant. Phenol (pH 8.0)–chloroform–isoamyl alcohol (25:24:1) (Beijing Dingguo Changsheng Biotechnology Co, Ltd., Beijing, China) mixture was added to the tube, inverted and mixed well, and then centrifuged again at 12,000 rpm for 10 min to obtain the supernatant. Next, chloroform–isoamyl alcohol (24:1) was added, followed by centrifugation at 12,000 rpm for 10 min to obtain the supernatant, which was transferred into a 1.5 mL centrifuge tube. Isopropanol was added, mixed by shaking, and precipitated at −20 °C. After centrifugation at 12,000 rpm for 10 min, the precipitate was washed twice with 1 mL of 75% ethanol (Beijing Dingguo Changsheng Biotechnology Co, Ltd., Beijing, China). Following another centrifugation, the supernatant was discarded, and the DNA pellet was dissolved in ddH_2_O (Beijing Dingguo Changsheng Biotechnology Co, Ltd., Beijing, China). After the DNA sample dissolved, 1 µL of RNase A (Beijing Dingguo Changsheng Biotechnology Co, Ltd., Beijing, China) was added to digest RNA, and the sample was incubated at 37 °C for 15 min. Primers V34F (CCTAYGGGGRBGCASCAG) and V34R (GGACTACNNGGGTATCTAAT) were used for the PCR amplification of the v34 region of the bacterial 16S rRNA gene. The reaction conditions involved the addition of 15 µL Phusion^®^ High-Fidelity PCR Master Mix (New England Biolabs, Ipswich, MA, USA), 0.2 µM primers, and a 10-ng genomic DNA template. PCR was performed with an initial denaturation step at 98 °C for 1 min, followed by 30 cycles of denaturation at 98 °C for 10 s, annealing at 50 °C for 30 s, and extension at 72 °C for 30 s. Finally, the reaction was maintained at 72 °C for 5 min [43,44]. The resulting PCR products were purified using magnetic beads, and samples of equal volume were mixed based on their concentrations. After thorough mixing, the PCR product was analyzed using an Agilent 5400 system(Agilent Technologies Inc., Santa Clara, CA, USA), and the target 250 bp fragment band was recovered. Subsequently, a library was constructed through a series of steps, including end repair, A-tailing, the addition of sequencing adapters, and purification. Upon verification of its quality using Qubit and Q-PCR quantification, the qualified library was submitted to Beijing Novogene Technology Co., Ltd. (Beijing, China) for PE250 sequencing using the NovaSeq6000 platform.

### 2.3. Control of Data Quality

Following sequencing, the barcode and primer sequences were truncated, followed by assembly of the reads for each sample using FLASH (version 1.2.11; http://ccb.jhu.edu/software/FLASH, accessed on 10 July 2024) [45], resulting in Raw Tag data (Raw Tags). Cutadapt software (Version 4.9) was used to match the reverse primer sequences and trim off the remaining sequences. Subsequently, Fastp software (version 0.23.1) was employed to rigorously filter the concatenated Raw Tags, yielding high-quality tag data (Clean Tags) [46]. The Tag sequences were annotated using the Silva database (https://www.arb-silva.de/, accessed on 10 July 2024) for the 16S rRNA. Chimeric sequences were identified and removed to obtain the final effective data (Effective Tags) [47].

### 2.4. Amplitude Sequence Variant Denoising and Species Annotation

For the acquired Effective Tags, the DADA2 module within QIIME2 software (Version QIIME2-202202) was used for denoising, yielding the final Amplitude Sequence Variants (ASVs) and feature table [48,49]. Species annotation was then conducted using the QIIME2 classification sklearn algorithm along with the Silva 138.1 database [50,51]. Based on the ASV annotation results and characteristic tables of each sample, we identified the top 10 species with the highest abundance for each sample across different taxonomic ranks (phyla, genera, and species) [31]. We used the SVG function in Perl to create a bar chart depicting the distribution of relative abundance. Additionally, we selected the top 100 genera with the highest abundance in the samples and plotted a phylogenetic tree in the SVG format using Perl to further investigate the phylogenetic relationships among the species at the genus level.

### 2.5. Statistical Analyses

#### 2.5.1. Analysis of Alpha Diversity

Alpha diversity was used to analyze the bacterial community diversity within the sample. QIIME2 software was used to calculate the chao1, dominance, goods coverage, observabd_otus, Pielou-e, Shannon, and Simpson indices [31]. The R value of the Plyr package (Version 1.8.9) was used to plot the dilution curve and to determine the appropriate sequencing quantity. The R package was used to draw a visualized species accumulation box plot to evaluate the richness and sample size of the microbial community [52].

#### 2.5.2. Analysis of Beta Diversity

To assess the complexity of community composition and compare differences among samples (or groups), β diversity analysis was conducted in QIIME2, utilizing both weighted and unweighted distances as the basis. First, based on the species annotation results of all samples and the abundance information of feature sequences, the feature sequence information of the same classification was consolidated to produce a species abundance profiling table. Simultaneously, the unweighted UniFrac distance was calculated by leveraging the phylogenetic relationships among feature sequences. Utilizing the abundance information of these feature sequences, a Weighted UniFrac distance was subsequently constructed to generate a distance matrix heatmap, which served as a measure of the dissimilarity coefficient between two samples. Using the weighted UniFrac distance matrix as a foundation, the UPGMA.tre function in QIIME was used to create a UPGMA clustering tree and examine the similarities among various samples. Principal Coordinate Analysis was conducted using the ade4 and ggplot2 packages in R software (version 4.0.3) to compute and visualize similarities in species composition and structure [30,53].

#### 2.5.3. Analysis of Community Differences and Functional Prediction Between Samples

Using the R package (Version 3.5.3), we conducted a *t*-test analysis with the significance level set at *p* < 0.05 to identify the species exhibiting significant differences between groups at the taxonomic level. The Phylogenetic Investigation of Communities by Reconstruction of Unobserved States 2 (PICRUSt2, V2.3.0) bioinformatics software package was used to predict the metagenomic functions of the marker genes and perform functional annotation of the sequencing data based on the Kyoto Encyclopedia of Genes and Genomes database. Using the annotation results, we selected the top 10 functions with the highest abundance at each annotation level for each sample and generated a stacked bar chart of functional relative abundance to visually represent the functions with higher relative abundance and their proportions at the phylum and genus annotation levels across the samples.

## 3. Results

### 3.1. Results of Data Quality Control

Sixteen samples were sequenced in the v3–v4 region of the 16S rRNA, yielding 1,615,038 Raw Tags. Following the assembly, 1,598,032 tag sequences were obtained. After the chimeric sequences were removed, 1,461,384 Effective Tags remained, with an average length of 419 nt. The GC base content was 53.27%, and Q20% and Q30% exceeded 98% and 94%, respectively (Supplementary Table S1). These results indicate the high quality and suitability of the v3–v4 sequencing data of the 16S rRNA for subsequent analysis.

### 3.2. Annotation Results of ASVs

**Figure 1:**
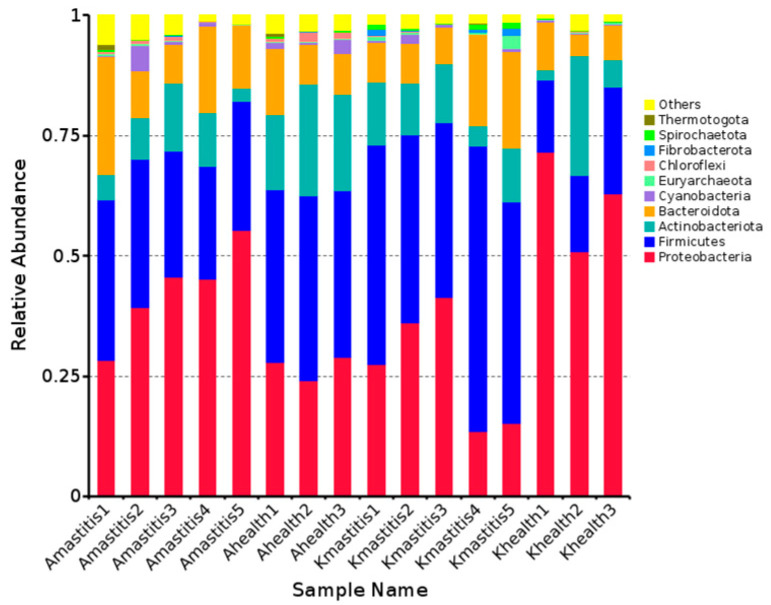
Relative abundance of gate-level species in subclinical mastitis versus healthy camel milk: top 10 histograms. The horizontal axis (Sample Name) represents the sample name, the vertical axis represents the relative abundance, and “others” refers to sum of the relative abundances of all gates except for the 10 gates in the graph.

The results indicated that 15,916 species from 16 camel milk samples were annotated to 49 phyla within the bacterial communities. In healthy Bactrian camel milk from Jimunai County, 40 bacterial phyla were annotated, predominated by *Firmicutes* (12.41%) and *Proteobacteria* (9.31%). In the milk of Bactrian camels with subclinical mastitis from the same county, 45 bacterial phyla communities were annotated, with *Proteobacteria* (44.40%) and *Firmicutes* (29.05%) being the most abundant, followed by phyla such as *Bacteroidota* (15.14%), *Actinobacteriota* (8.71%), and *Cyanobacteria* (1.45%). In the milk of healthy Bactrian camels from Keping County, 34 bacterial phyla were annotated, predominated by *Proteobacteria* (63.05%) and *Firmicutes* (17.97%). Thirty-three bacterial phyla were annotated in the milk of camels with subclinical mastitis from the same county, among which *Proteobacteria* (27.29%) and *Firmicutes* (46.03%) were the most abundant. Other notable phyla included *Bacteroidetes* (12.83%), *Actinobacteria* (10.39%), and *Euryarchaeota* (1.02%). Bar graph analysis of species abundance revealed that in comparison to healthy camel milk, milk from Bactrian camels with subclinical mastitis from Jimunai County exhibited increased abundance of both *Proteobacteria* and *Firmicutes* at the phylum level. Conversely, in Bactrian camels from Keping County, the abundance of *Proteobacteria* decreased, whereas that of *Firmicutes* increased (Figure 1 and Supplementary Table S2). The phyla *Marinimicrobia*, *Nanoarchaeota*, and *Halanaerobiaeota*, were absent in camel milk from of Jimunai County, whereas *Sumerlaeota* and *Abditibacteriota*, along with nine other bacterial phyla (including *Entotheonellaeota*) were not detected in camel milk of Keping County. Thus, at the phylum level, the microbial community structure of Bactrian camel milk from Jimunai County was more complex than that from Keping County.

**Figure 2:**
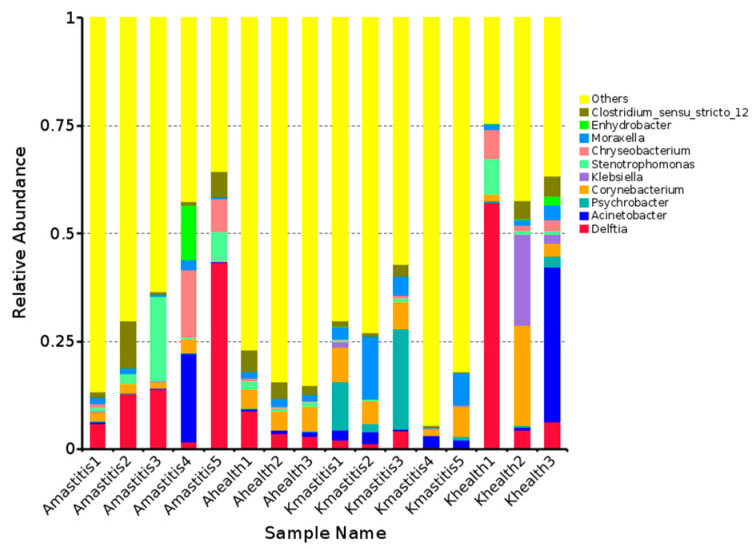
Relative abundance of the top 10 bacterial genera in milk from camels with subclinical mastitis versus healthy camels. The horizontal axis represents the sample names and the vertical axis indicates the relative abundance. “Others” signifies the combined relative abundance of all other genera not included among the ten genera depicted in the graph.

At the genus level, 1060 bacterial genera were annotated in camel milk. The bacterial community in healthy Bactrian camel milk from Jimunai County comprised 621 genera, dominated by *Delftia* (30.19%) and *Corynebacterium* (28.30%). In contrast, the bacterial community in the milk of Bactrian camels with subclinical mastitis from the same county comprised 776 genera, predominated by *Delftia* (38.53%), followed by *Stenotrophomonas* (14.85%), *Chryseobacterium* (11.94%), *Acinetobacter* (10.45%), and *Clostridium* (9.45%). The bacterial community in the milk of healthy Bactrian camels from Keping County comprised 497 genera, predominantly consisting of *Psychrobacter* (30.08%) and *Moraxella* (10.25%). In contrast, the bacterial community in the milk from camels with subclinical mastitis in Keping County encompassed 590 genera, with *Psychrobacter* (30.28%), *Moraxella* (23.57%), *Corynebacterium* (22.76%), *Acinetobacter* (8.13%), and *Delftia* (6.50%) being the most prominent (Figure 2 and Supplementary Table S3).

In addition, bacterial communities belonging to 153 genera, including *Succinivibrionaceae*, *Sulfitobacter*, and *Pasteurella*, were absent in the milk of Bactrian camels from Jimunai County, whereas 357 genera, including *Vulcaniibacterium, Schlegelella*, and *Chelatococcus*, were not detected in the milk of camels from Keping County. This discrepancy may be attributed to differences in breeding environments. Specifically, Bactrian camels in Keping County were reared in a relatively homogeneous environment with excellent management, disinfection, and disease prevention measures. In contrast, Bactrian camels from Jimunai County were exposed to more diverse living environments, including barns, the Gobi Desert, and grasslands, leading to significantly higher bacterial diversity in their milk than that of camels from Keping County.

**Figure 3:**
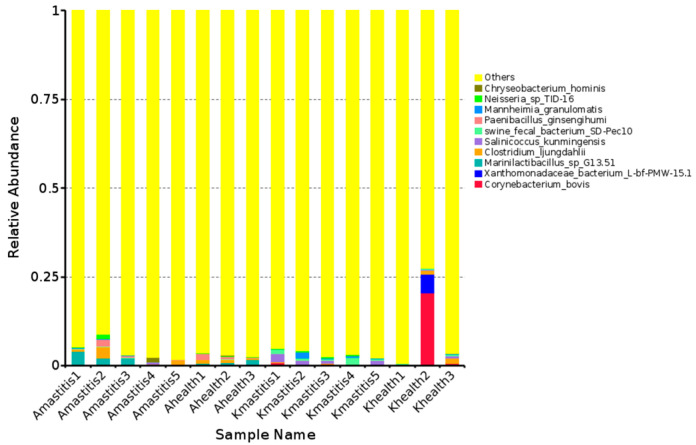
Relative abundance of the top 10 bacterial species in milk from camels with subclinical mastitis versus healthy camels. The horizontal axis represents the sample name, the vertical axis represents the relative abundance, and “others” refers to the sum of the relative abundances of all species, except for the 10 species in the graph.

**Figure 4:**
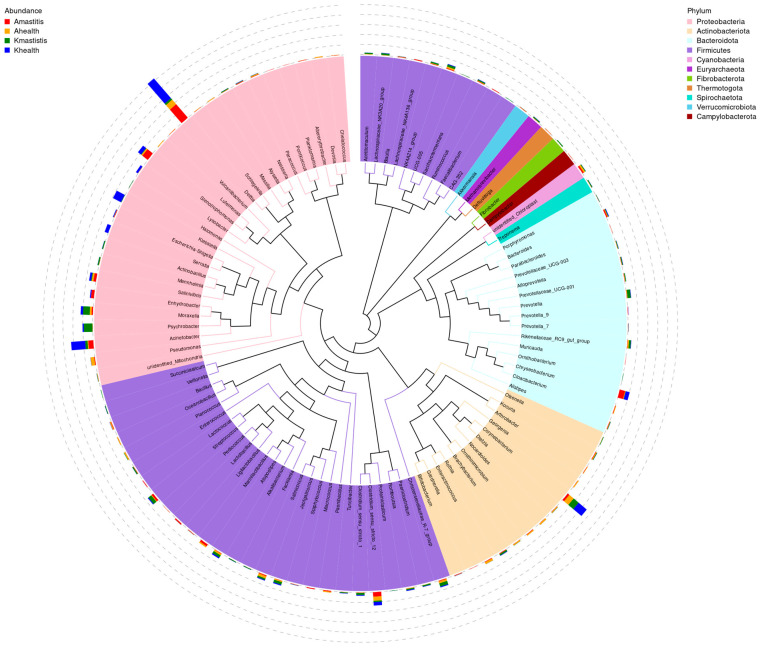
Genus-level phylogenetic relationships between subclinical mastitis and healthy camel milk. The colors of the branches and sectors indicate their respective corresponding phyla, whereas the stacked bar chart positioned on the outer side of the sector ring displays the abundance of the bacterial genera across various samples. The legend on the left specifies the samples and the legend on the right specifies taxonomic classifications at the phylum level which correspond to those at the genus level.

Overall, 788 bacterial species were identified in camel milk. In healthy Bactrian camel milk from Jimunai County, 248 bacterial species were identified, predominated by *Marinilactibacillus*, *Clostridium ljungdahlii*, and *Paenibacillus ginsengihumi*, accounting for 33.33, 26.67, and 22.22%, respectively. In the milk of Bactrian camels with subclinical mastitis from the same county, 341 bacterial species were detected, with *Marinilactibacillus, Clostridium ljungdahlii*, and *Paenibacillus ginsengihumi* being the most abundant, accounting for 42.86, 28.57, and 12.38%, respectively. The onset of subclinical mastitis in Bactrian camels from Jimunai County was accompanied by an increase in the diversity and abundance of bacterial species in their milk. The milk of healthy Bactrian camels from Keping County harbored 232 bacterial species, predominantly *Corynebacterium bovis* (70.33%), *Xanthomonadaceae* bacteria (1.78%), and *Clostridium ljungdahlii* (0.83%). In contrast, milk from camels with subclinical mastitis contained 292 bacterial species, with *Salinicoccus kunmingensis* (31.94%), swine fecal bacteria (30.06%), and *Mannheimia granulomatis* (16.47%) being the most prevalent (Figure 3). Notably, the onset of subclinical mastitis in Bactrian camels from Keping County resulted in increased and altered bacterial diversity in camel milk. Among the top 10 most abundant bacterial species, neither *Corynebacterium bovis* nor *Xanthomonadaceae* bacteria were detected in the milk of camels from Jimunai County, while *Paenibacillus ginsengihumi* was absent in the milk of camels from Keping County. The phylogenetic tree indicated that genera with closer phylogenetic ties were predominantly clustered within 11 phyla, including *Proteobacteria*, *Firmicutes*, and *Actinobacteria*. Notably, genera such as *Chrisensenellaceae*, *Campylobacter*, *Olsenella*, and *Paenibacillus* were relatively independent (Figure 4 and Supplementary Table S4).

### 3.3. Analysis of Bacterial Abundance and Diversity in Camel Milk Samples in Healthy and Subclinical Mastitis Conditions

**Figure 5:**
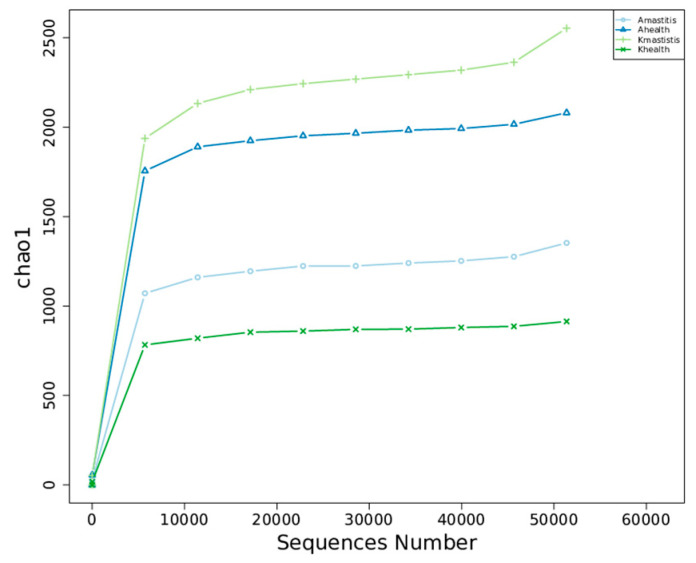
Dilution curves of species in the milk from subclinical mastitis and healthy camelids.

**Figure 6:**
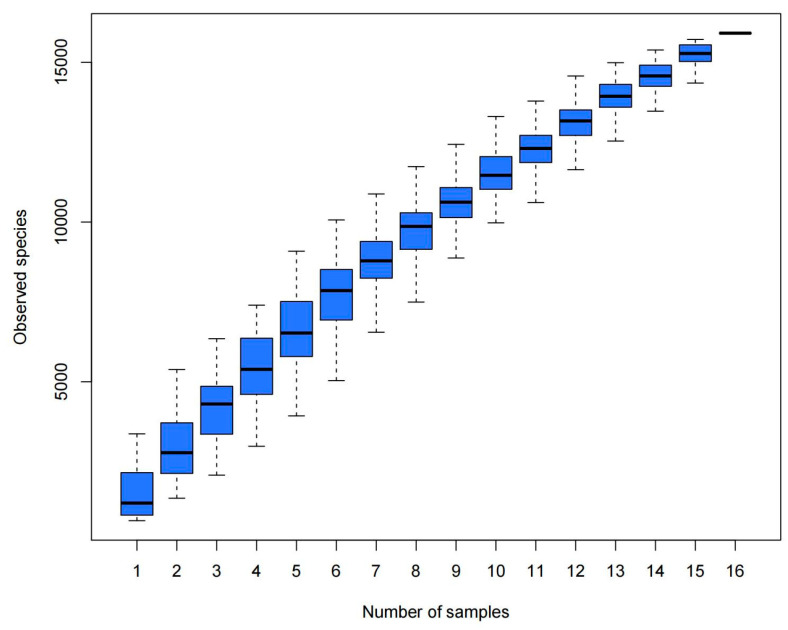
Cumulative box plot of alpha diversity species.

**Figure 7:**
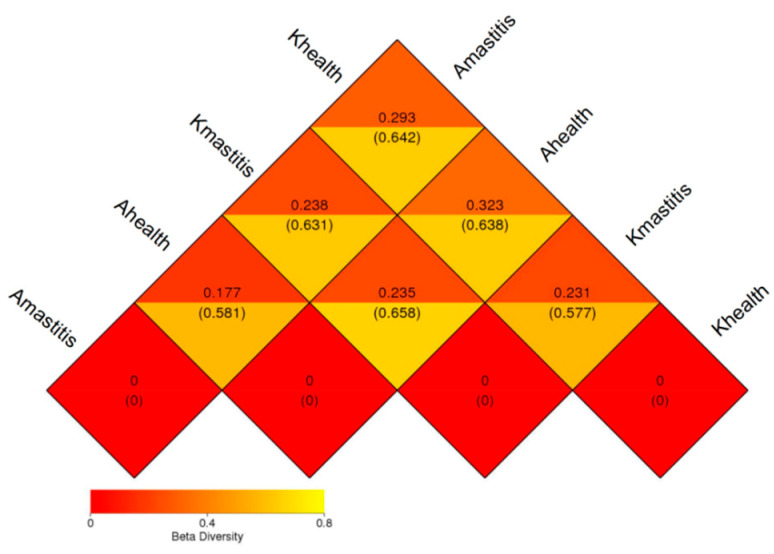
Heat map of the Beta diversity index (UniFrac distance matrix) between subclinical mastitis and healthy camel milk. The numbers in the squares of the figure represent the dissimilarity coefficients between the pairs of samples. The smaller the dissimilarity coefficient, the lesser the difference in species diversity between the two samples. Within the same square, the upper and lower values represent the weighted and unweighted UniFrac distances, respectively.

**Figure 8:**
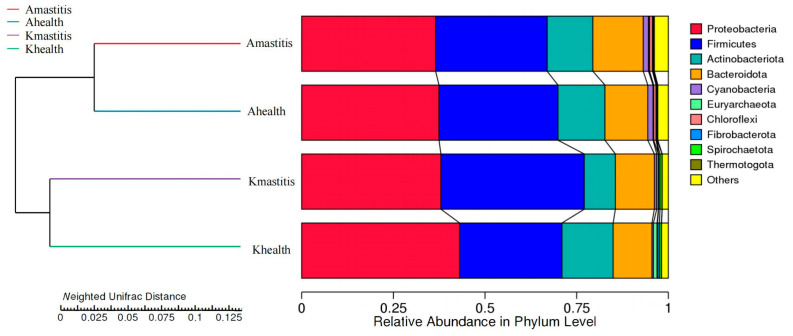
UPGMA clustering tree at the phylum level based on weighted UniFrac distances between subclinical mastitis and healthy camel milk. The UPGMA clustering tree structure is shown on the left, whereas the graph on the right depicts the relative abundance distribution for each sample at the phylum level.

**Figure 9:**
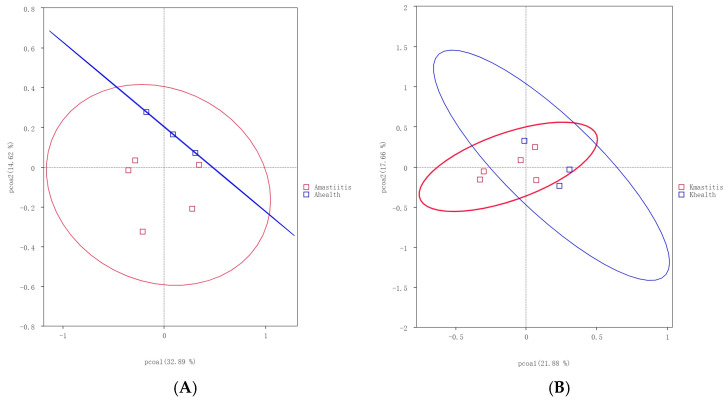
PCoA analyses comparing subclinical mastitis and healthy milk samples in Jimunai County (**A**) and Keping County (**B**). The horizontal axis represents one principal component, the vertical axis represents another principal component, and the percentage represents the contribution value of the principal component to the sample difference. Each point in the figure represents a sample, and samples in the same group are represented by the same color.

Alpha diversity analysis revealed that the average Chao1 index in healthy Bactrian camel milk from Jimunai County was 1980.31, with 1964 observed features. In Bactrian camel milk from the same county with subclinical mastitis, the average Chao1 index was 1240.69, with 1217 observed features. For healthy Bactrian camel milk from Keping County, the average Chao1 index was 873.22, with 873 observed features. Conversely, in Bactrian camel milk from Keping County with subclinical mastitis, the average Chao1 index was 2269.09, with 2296 observed features (Supplementary Table S5). Bactrian camels from Jimunai County exhibited reduced bacterial richness in milk on being infected with subclinical mastitis, whereas increased abundance was observed in camel milk from Keping County. Moreover, the dilution curves for both healthy and subclinical mastitis-affected milk samples gradually plateaued, suggesting that the sequencing volume was adequate to encompass the majority of the microbial taxa. An increase in sequencing volume may yield only a small number of novel species, indicating that the sequencing data volume was reasonable (Figure 5). Additionally, gradual flattening of the box plot positions suggested that the species composition of camel milk did not significantly increase with an expanded sample size, thereby facilitating data analysis (Figure 6). Based on the Beta Diversity Index UniFrac Distance Matrix heatmap (Figure 7), camel milk samples exhibited a substantial coefficient of dissimilarity, suggesting variations in species diversity among the Bactrian camel milk samples sourced from Jimunai County and Keping County. Analysis of the UPGMA clustering tree revealed that bacterial communities such as *Proteobacteria*, *Firmicutes*, *Actinobacteria*, and *Bacteroidetes* were relatively abundant in camel milk at the phylum level. Notably, variations in *Proteobacteria* could visually delineate the differences between subclinical mastitis and healthy camel milk (Figure 8). Principal coordinate analysis revealed that healthy and subclinical mastitis samples of Bactrian camels from Jimunai County exhibited a relatively high degree of clustering, suggesting a significant similarity in their community structure. Nevertheless, a healthy sample from Keping County’s Bactrian camel population nearly coincided with a subclinical mastitis sample, potentially owing to variations in the CMT detection method (Figure 9).

The horizontal axis represents the amount of sequencing data and the vertical axis represents the corresponding alpha diversity indices.

The abscissa represents the sample size, and the ordinate represents the number of feature sequences after sampling.

### 3.4. Analysis of Community Differences Among Samples and Functional Prediction

**Figure 10:**
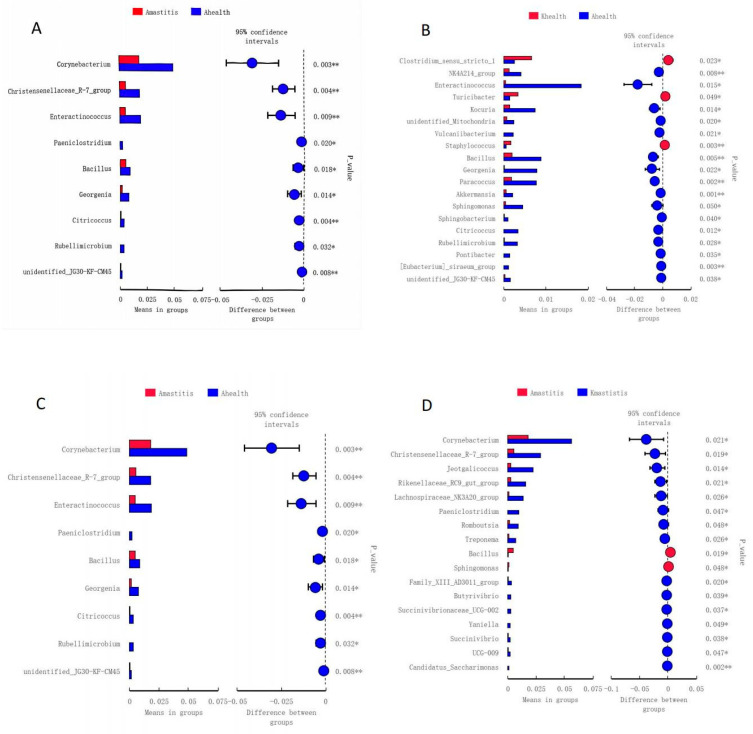
Analysis of species differences between subclinical mastitis and healthy milk using *t*-test groups. Panels (**A**,**B**) demonstrate the differential analysis of healthy and mastitis-infected Bactrian camels from Jimunai County and Keping County, respectively. Panels (**C**,**D**) show the differential analyses of milk from healthy and mastitis-infected camels, respectively. The left sides of the panels display the abundances of significantly different species between groups. Each bar in the graph represents the mean of significantly different abundant species between groups in each group. The right sides of the panels display inter-group confidence levels. The extreme left and right points of each circle in the panels represent the lower and upper limits of the 95% confidence interval for the mean difference, respectively. The center of the circles signifies the difference in means, while the circle’s color indicates the *p*-value from the significant difference test between groups for the corresponding species, * indicates *p* value < 0.05, ** indicates *p* value < 0.01.

**Figure 11:**
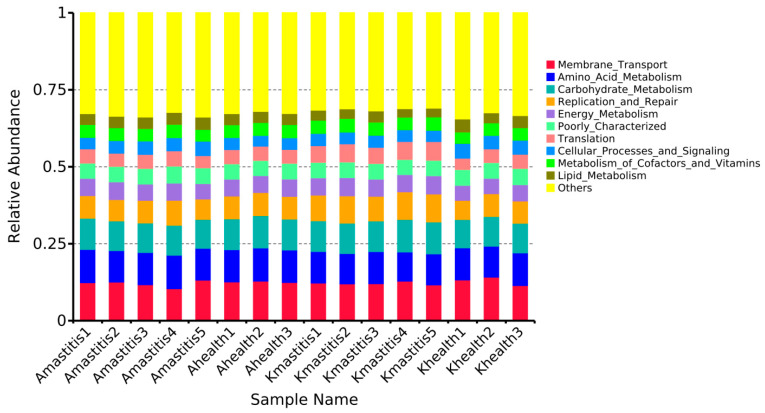
PICRUSt2 functionally annotated relative abundance histogram of subclinical mastitis versus healthy milk samples. The horizontal axis represents the sample names; the vertical axis indicates the relative abundance; and “Others” signifies the sum of the relative abundances of all other functional information beyond the 10 shown in the figure.

The *t*-test gate-level analysis revealed significant differences in the abundances of bacteria such as *Corynebacterium*, *Christensenellaceae*, and *Enterococcus*, between the healthy and subclinical mastitis milk samples of Bactrian camels from Jimunai County (Figure 10A). When comparing milk samples from healthy and subclinical mastitis-infected Bactrian camels in Keping County, notable differences were observed in the abundances of *Christensenellaceae*, *Salinivibrio*, and *Jeotgalicoccus* species (Figure 10B). Bacterial community abundance in milk from healthy Bactrian camels in Jimunai County was higher than that in milk from healthy camels from Keping County, particularly differing in the abundance of *Enterococcus*, which was considerably higher in healthy milk from Bactrian camels in Jimunai County than that in milk from Keping County (Figure 10C). When subclinical mastitis occurred, notable differences emerged in the abundance of bacteria such as *Corynebacterium*, *Christensenellaceae*, and *Jeotgalicoccus* in the milk of Bactrian camels from Jimunai County versus those from Keping County, with markedly higher bacterial community abundance in milk from camels of Keping County (Figure 10D). According to the relative abundance analysis of PICRUSt2 functional annotations, functions such as Membrane Transport, Amino Acid Metabolism, and Carbohydrate Metabolism were decreased in Bactrian camels with subclinical mastitis (Figure 11).

## 4. Discussion

In China, with improved quality of life, the quality requirements for camel milk, particularly the microbial composition, have also increased [34]. The complex microbial composition of camel milk originates from the environment and diseases, among which subclinical mastitis primarily affects camel milk quality, and bacteria are the main cause of subclinical mastitis in camels [54,55,56]. Cultivation-based microbial analysis cannot effectively identify important foodborne pathogens in milk. With the widespread application of high-throughput sequencing for milk sourced from humans, donkeys, cows, and unimodal camels, the microbial population composition has gradually been revealed [39,57,58,59]. However, research on the pathogens causing subclinical mastitis in Bactrian camels is lacking. This study involved the use of 16S rRNA gene amplicon sequencing to explore the impact of alterations in bacterial communities within camel milk on subclinical mastitis in Bactrian camels from Jimunai County and Keping County.

The results revealed that bacterial communities in healthy camel milk from Jimunai County and Keping County consisted of 40 phyla and 34 phyla, respectively, whereas those in camel milk with subclinical mastitis comprise 45 phyla and 33 phyla, respectively, which were significantly higher than those in healthy and mastitis camel milk (26 phyla and 16 phyla, respectively). Bacterial communities at the phylum level in milk from mastitis-infected dromedary and Bactrian camels are lesser than those in healthy camel milk [31]. The bacterial community in the milk of healthy, captive-bred Bactrian camels in Inner Mongolia comprises 32 phyla [38], closely resembling the bacterial diversity observed in milk from healthy Bactrian camels from Keping County, as assessed in this study. This further confirms that variations in breeding practices are the primary factors influencing differences in bacterial communities in camel milk [39]. The findings of this study revealed that *Firmicutes*, *Proteobacteria*, *Bacteroidetes*, and *Actinobacteria* were the dominant microbiota in both subclinical mastitis and healthy milk samples, suggesting a significant correlation between the microorganisms present in healthy and diseased camel milk [60]. The bacterial diversity in the milk of Bactrian camels is similar to those of animals such as dromedary camels, Sahiwal cattle, buffaloes, Holstein cows, and donkeys [30,31,58,59,61]. Mastitis in dromedary camels results in an increase in the abundance of Firmicutes and a decrease in the abundance of *Proteobacteria* [31]. These changes were observed in the microbiota of milk from Bactrian camels with subclinical mastitis in Keping County. Notably, Bactrian camels with subclinical mastitis from Jimunai County exhibited an increase in the abundance of both *Proteobacteria* and *Firmicutes* at the phylum level, suggesting disrupted breast tissue structure and milk homeostasis, with rapid alterations in bacterial abundance contributing to the onset of mastitis [58]. *Proteobacteria* and *Firmicutes* are ubiquitous in environments such as soil, the intestinal tract, and milk [62,63,64], and can cause various diseases. Progressive studies suggest that altered *Proteobacteria* proportions may indicate disease onset [65], while also facilitating lipopolysaccharide biosynthesis and carbohydrate metabolism. Conversely, *Firmicutes* harbor genes associated with cell wall biosynthesis and membrane transport processes [63].

At the genus level, the bacterial community abundance was higher in Jimunai camel milk than in Keping camel milk. Subclinical mastitis resulted in significantly increased bacterial diversity in milk. *Delftia* and *Corynebacterium* were predominant in Jimunai camel milk, *Psychrobacter* and *Moraxella* predominated in Keping camel milk, and *Glutamicibacter* and *Schlegelella* were predominant in healthy camel milk, while *Streptococcus* and *Schlegelella* were predominant in mastitis camel milk [31]. These findings further confirmed that rapid change in bacterial abundance induced the occurrence of mastitis [58]. Healthy Bactrian camel milk from Inner Mongolia predominantly comprises species such as *Pseudomonas*, *Thermus*, and *Streptococcus* [38], whereas healthy Bactrian camel milk from Xinjiang primarily includes genera such as *Epilithonimonas*, *Klebsiella*, and *Kluyveras* [36], highlighting regional variations in bacterial diversity within camel milk. The presence of *Delftia* has been documented in milk sourced from humans, cows, and donkeys, emerging as a potential source of multidrug resistance in fresh milk [66,67,68]. Whether *Delftia*, as reported for the first time in camel milk, is related to multidrug resistance remains to be studied. According to the PATRIC database, the genome of *Corynebacterium* has been reported in 83 species and is recognized as an important pathogen causing zoonotic diseases [69]. Several species such as *Corynebacterium diphtheriae*, *Corynebacterium ulcerans*, and *Corynebacterium pseudotuberculosis* have been identified in human diseases, causing conditions like diphtheria, tracheitis, pharyngitis, sinusitis, and bronchitis [70]. In animals, species such as *Corynebacterium amycolatum*, *Corynebacterium Aquilae*, *Corynebacterium auriscanis*, and *Corynebacterium bovis* are associated with caseous lymphoid infections in ruminants [71]. Additionally, *Psychobacter* and *Moraxella* are the pivotal pathogenic bacteria implicated in respiratory syndromes in calves [72]. Nevertheless, to our knowledge, no cases of animal mastitis caused by *Psychobacter* have been reported to date [30]. In Xinjiang, local herders prefer to consume fresh camel milk [36]. However, when Bactrian camels suffer from subclinical mastitis, alterations occur in the quality and bacterial microbiota of the camel milk, and the presence of drug-resistant pathogens in the milk could potentially pose risks to human health [73]. Subclinical mastitis in camels from Jimunai County did not alter the predominant bacteria types, but rather significantly altered the abundance of relevant pathogenic bacteria. Alterations in the abundance of bacteria, such as *Marinilactibacillus*, *Clostridium ljungdahlii*, and *Paenibacillus ginsengihumi*, may be the primary cause of subclinical mastitis in Bactrian camels from Jimunai County. Notably, *Marinilactibacillus* and *Clostridium ljungdahlii* are associated with metabolism and fermentation, potentially owing to the frequent fermentation observed in freshly stored Jimunai camel milk catalyzed by these bacteria [74,75]. Unlike Bactrian camels from Jimunai County, the primary bacteria involved in subclinical mastitis among Bactrian camels in Keping County were *Salinicoccus kunmingensis*, swine fecal bacteria, and *Mannheimia granulomatis*. Subclinical mastitis induces a shift in bacterial species, relative to that in healthy milk. The presence of *Salinicoccus kunmingensis* has been commonly reported in the milk of dromedary camels in Saudi Arabia; it poses significant risks not only to Bactrian camels but also to humans consuming unheated or unpasteurized fresh milk [76]. Swine fecal bacteria are mainly found in pig feces [77]; however, their presence in camel milk, owing to cross-species transmission, remains to be elucidated. *Mannheimia granulomatis* is found in the nasopharynx of healthy dromedary camels in northern Kenya, and is an important pathogen in both domesticated and wild ruminants [78].

Based on the alpha diversity index and dilution curve results, the bacterial abundance in healthy Bactrian camel milk from Jimunai County was notably higher than that in milk from camels with subclinical mastitis. These findings align with those of previous studies on mastitis in dromedary camels and water buffaloes [31,61]. Nevertheless, in Keping County, an increase in bacterial abundance was observed in milk from Bactrian camels with subclinical mastitis, a phenomenon attributable to factors such as farming practices, breeding environment, and geographical location. Among all the milk samples tested, bacterial abundance ranked as follows: subclinical mastitis milk from Bactrian camels in Keping County, healthy milk from Bactrian camels in Jimunai County, subclinical milk samples from Bactrian camels in Jimunai County, and healthy milk from Bactrian camels in Keping County. Variations in bacterial abundance indicate a direct correlation between the occurrence of subclinical mastitis in Bactrian camels and their breeding environments [31]. The beta diversity index revealed significant differences in milk sample diversity between Bactrian camels from Jimunai County and Keping County, and the results of the UPGMA cluster tree analysis were consistent with the ASV annotations at the phylum level.

Analysis of the relative abundance based on PICRUSt2 functional annotation revealed alterations in the abundance of functions, such as membrane transport, amino acid metabolism, and carbohydrate metabolism, during subclinical mastitis in Bactrian camels. After the camel’s mammary gland is infected by microorganisms, the innate immune system first recognizes and then triggers persistent adaptive immunity, which initiates the innate immune response and inflammatory response by recognizing the microbial-associated molecular pattern through the pattern recognition receptor. On reaching the mammary gland, bacteria proliferate and produce toxins; the local chemical penetration attracts phagocytes to clear pathogens, and the T cells in milk produce adaptive immune responses to clear the infection. The transmembrane transport of breast cells plays an important role in this process [19,79,80,81], which also explains why PICRUSt2 function annotation is predominantly focused on membrane transport in cases of subclinical mastitis in Bactrian camels. Amino acids, which serve as constituents of protein synthesis, are essential cellular structural components and energy sources for normal cell growth, differentiation, and function. Disorders in amino acid metabolism are associated with various diseases [82], and the concentration of amino acids in milk correlates with the severity of mastitis [83]. Subclinical mastitis in cows induced by mastitis-associated coagulase-negative *Staphylococcus* results in a significant decrease in the concentrations of tryptophan, kynurenine, and kynurenic acid in their milk [84]. Due to the impact of subclinical mastitis, cows may exhibit the activation of innate immunity and altered carbohydrate metabolism several weeks prior to diagnosis. When cows are diagnosed with subclinical mastitis prior to parturition, they exhibit higher levels of serum lactate, serum amyloid A, tumor necrosis factor, and lactate [85]. Moreover, subclinical mastitis can be predicted by changes in amino acids and carbohydrates at 8 and 4 weeks before delivery [86]. However, the occurrence of similar changes in Bactrian camels requires further research.

A limitation of this study is that the sample size collected from the two regions was relatively small, with only 16 milk samples in total. The main reason for this is that Bactrian camels, as reflex lactating animals, require stimulation by suckling calves before they can secrete milk [3]. This leads to generally low milk production in Bactrian camels, increasing the difficulty of sampling. In addition, it was necessary to ensure that the female camels used for sampling had the same parity and lactation period, with no medical history and no use of antibiotics, further reducing the number of samples collected in this study. Furthermore, the pathogen causing mastitis was not isolated or identified in this study, limiting its ability to provide effective guidance for precise prevention, control of mastitis, and antibody reduction strategies. As livestock with a relatively unique distribution, there is relatively less research on mastitis in Bactrian camels. Although the results of this study can reflect the pathogenesis of subclinical mastitis in Bactrian camels, they do not fully represent the entire Bactrian camel industry. In the future, research on mastitis in Bactrian camels should aim to understand the incidence of mastitis across different ages, parity, lactation periods, and breeding environments. It should also include some basic indicators such as milk composition, somatic cell count, blood biochemistry, blood routine, bacterial community, and pathogenic bacteria in camel milk when mastitis occurs, providing guidance for the rapid diagnosis and prevention of mastitis in Bactrian camels.

## 5. Conclusions

As the main breeding area for Bactrian camels in China, the healthy development of the Bactrian camel industry in Xinjiang is related to the adjustment of the agricultural and animal husbandry structure and the increase in income of farmers and herdsmen. However, the large-scale occurrence of subclinical mastitis in Bactrian camels has affected the healthy development of the Bactrian camel industry. This study used the v34 region amplicon sequencing technology of the 16S rRNA gene to investigate the changes in bacterial communities in the milk of Bactrian camels with subclinical mastitis in different regions. It was found that the occurrence of subclinical mastitis in Bactrian camels was related to changes in bacterial abundance and species in camel milk. This study clarifies the pathogenesis of subclinical mastitis in Bactrian camels in different regions, providing a reference for future pathogen research and prevention of subclinical mastitis in Bactrian camels.

## Data Availability

The datasets presented in this study can be found in online repositories. The names of the repository/repositories and accession number(s) can be found below: https://www.ncbi.nlm.nih.gov/bioproject/PRJNA1158948, accessed on 13 March 2025.

## Supplementary Materials

The following supporting information can be downloaded at https://www.mdpi.com/article/10.3390/microorganisms13061394/s1: Table S1: Statistics of data quality control results; Table S2: Relative abundance of species at the phylum level; Table S3: Relative abundance of species at the genus level; Table S4: Species-level relative abundance; Table S5: Alpha diversity index statistics.

## Abbreviations

The following abbreviations are used in this manuscript:

CMT	California Mastitis Test
PCR	Polymerase Chain Reaction
ASV	Amplitude Sequence Variant
PICRUSt2	Phylogenetic Investigation of Communities by Reconstruction of Unobserved States 2
CTAB	Cetyltrimethylammonium Bromide
UPGMA	Unweighted Pair Group Method with Arithmetic Mean
